# Supplementary material for: Synergistic Effects of Bacteriophage vB_Eco4-M7 and Selected Antibiotics on the Biofilm Formed by Shiga Toxin-Producing Escherichia coli
Source: Antibiotics (Basel). 2022 May 25;11(6):712. doi: 10.3390/antibiotics11060712 (PMC9219966; doi:10.3390/antibiotics11060712)
Supplement: Supplementary file 1 [file antibiotics-11-00712-s001.zip › antibiotics-1743318-supplementary.pdf]

Supporting Information

Synergistic effects of bacteriophage vB_Eco4-M7 and selected antibiotics on the biofilm formed by Shiga toxin-producing *Escherichia coli*

Agnieszka Necel [1,†], Sylwia Bloch [1,†], Gracja Topka-Bielecka [1], Agata Janiszewska [1], Aleksandra Łukasiak [1], Bożena Nejman-Faleńczyk [1,*,‡] and Grzegorz Węgrzyn [1,*,‡]

[1] Department of Molecular Biology, Faculty of Biology, University of Gdansk, Wita Stwosza 59, 80-308 Gdansk, Poland; agnieszka.necel@phdstud.ug.edu.pl (A.N.), sylwia.bloch@ug.edu.pl (S.B.), gracja.topka-bielecka@ug.edu.pl (G.T.-B.), a.janiszewska.477@studms.ug.edu.pl (A.J.), a.lukasiak.639@studms.ug.edu.pl (A.Ł.)

* Correspondence: bozena.nejman-falenczyk@ug.edu.pl (B.N.-F.);
grzegorz.wegrzyn@biol.ug.edu.pl (G.W.);
Tel.: +48 58 523 6024

† Co-firs author: These authors contributed equally to this work.

‡ Co-last author: These authors contributed equally to this work.

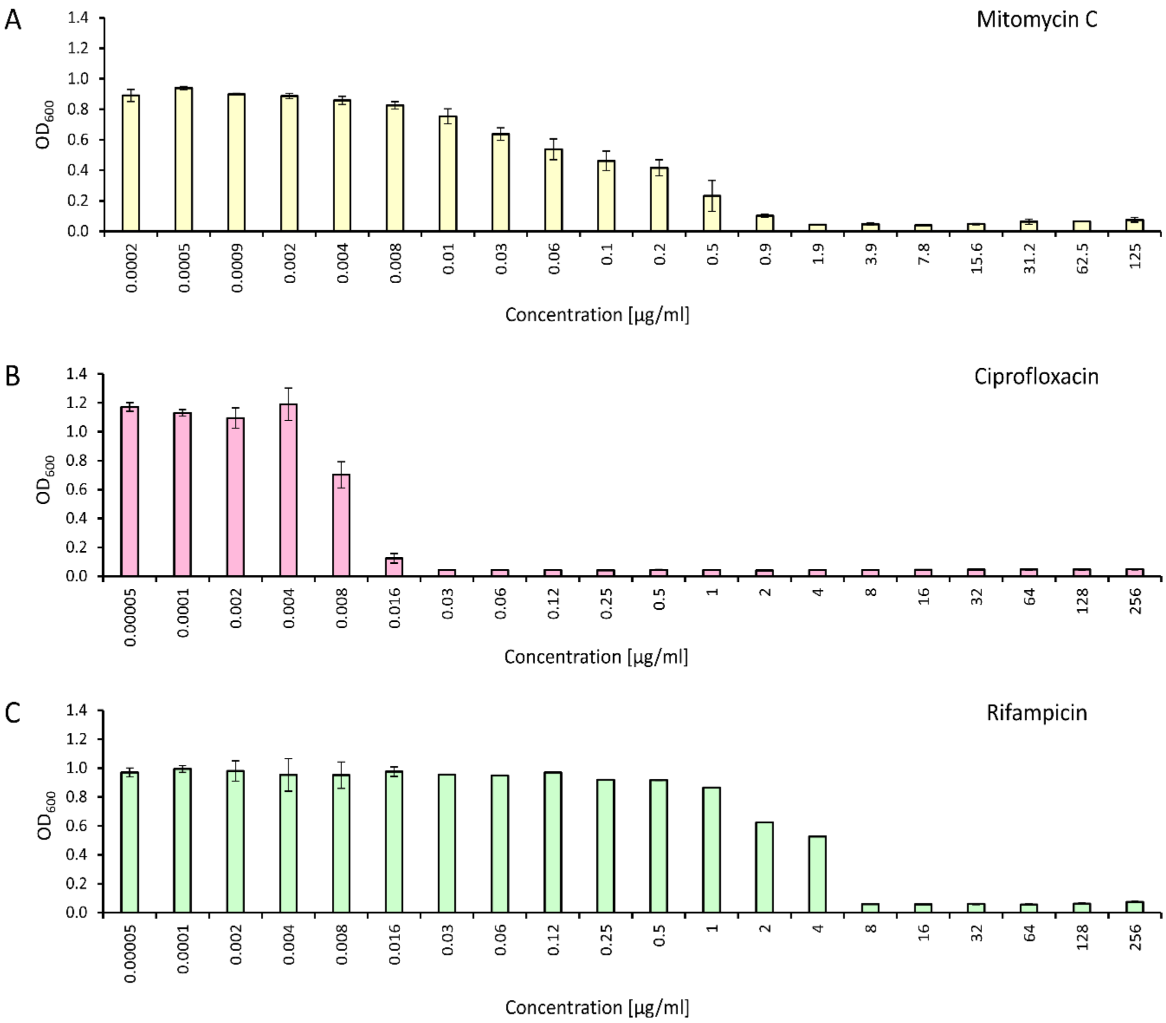

Figure S1. Effects of tested antibiotics on *E. coli* O157:H7 (ST2-8624) growth. Minimal inhibitory concentration (MIC) was determined for each antimicrobial agent: mitomycin C **(A)**, ciprofloxacin **(B)** and rifampicin **(C)**. The results are presented as OD_{600} of the bacterial culture measured after a 24-h incubation with the tested antibiotic. Mean values from three independent experiments are shown with error bars indicating SD. Note that in some cases the bars are smaller than the sizes of symbols.

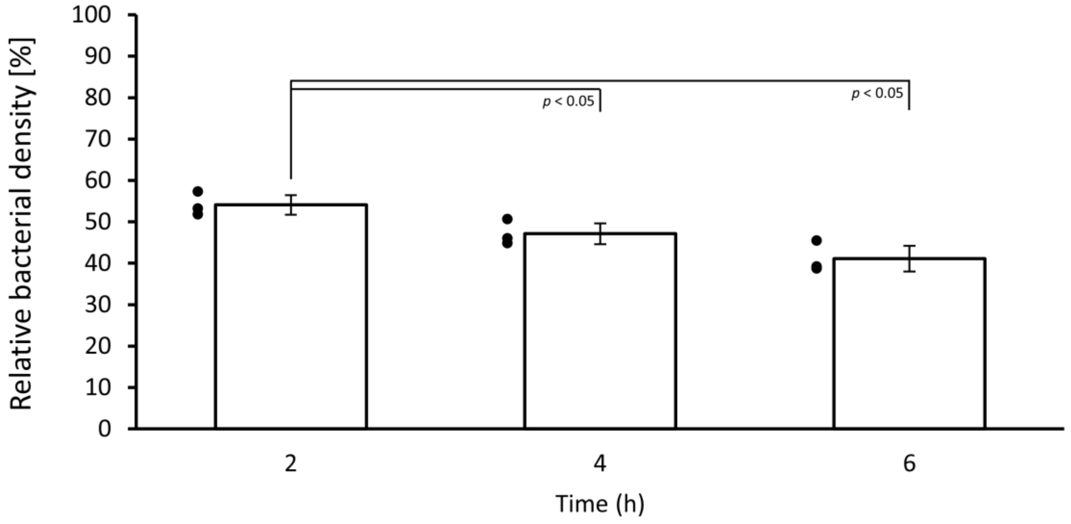

Figure S2. Bacterial biofilm density after 2-, 4- and 6-h incubation with the vB_Eco4M-7 phage alone, estimated by measuring the optical density at 600 nm. The data are presented relative to the control variant and show the significance at the level of $p < 0.001$, calculated using the one-way ANOVA. Statistical significance between particular time variants was calculated in the same way and is marked on the graph. The black dots next to the columns denote datapoints from individual experiments.

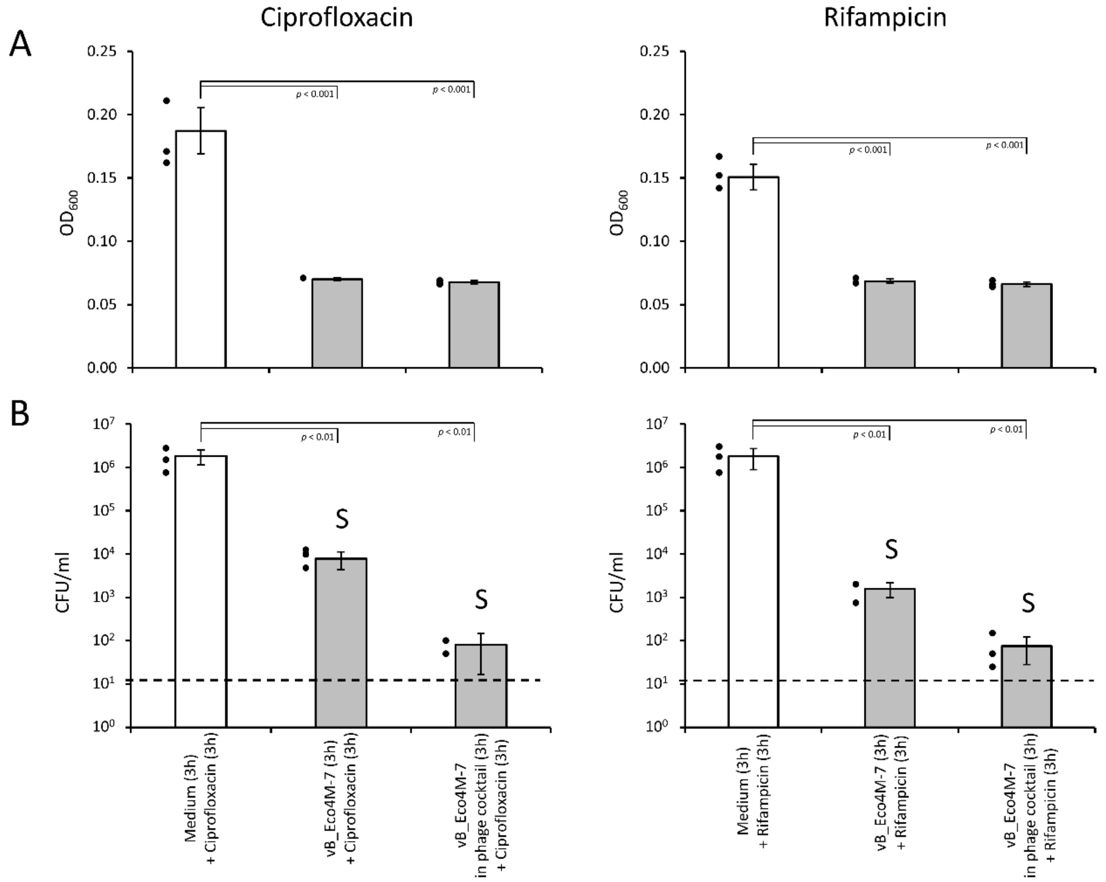

Figure S3. Bacterial biofilm density and viability of cells after a 6-h treatment with different antimicrobial agents added sequentially: (i) LB medium replaced after a 3-h incubation with ciprofloxacin (500 x MIC) or rifampicin (400 x MIC), (i) vB_Eco4M-7 phage (MOI of 100) replaced after a 3-h incubation with ciprofloxacin (500 x MIC) or rifampicin (400 x MIC), (iii) the phage cocktail (MOI of 100) mixed with ciprofloxacin (500 x MIC) or rifampicin (400 x MIC). The results were estimated by measuring the optical density at a wavelength of 600 nm **(A)** and enumerating the number of survivors per ml of the suspension (CFU/ml) **(B)**. Signatures of the X-axis are the same for all panels and are placed at the bottom of the figure for clarity. "S" indicates synergistic interaction of tested agents **(B)**. The black dashed lines represent the lower limit of detection **(B).** The results are presented as the mean values ± SD from three independent experiments. The black dots next to the columns denote datapoints from individual experiments. Statistical analyses were performed using the one-way ANOVA (p<0.05 was considered statistically significant).
